# Is Chlorhexidine an Ideal Vehicle for Calcium Hydroxide? A Microbiologic Review

**Published:** 2012-08-01

**Authors:** Zahed Mohammadi, Sousan Shalavi

**Affiliations:** 1. Department of Endodontics, Hamedan University of Medical Sciences, Hamedan, Iran; 2. Iranian Center for Endodontic Research, Shahid Beheshti University of Medical Sciences, Tehran, Iran; 3. Hamedan University of Medical Sciences, Hamedan, Iran

**Keywords:** Calcium Hydroxide, Candida Albicans, Chlorhexidine, Enterococcus Faecalis

## Abstract

Microorganisms play a major role in the initiation and perpetuation of pulpal and periapical disease. In order to predictably achieve a bacteria-free root canal system, it is necessary to use intracanal medicaments. Calcium hydroxide [Ca(OH)_2_] is the most common intracanal medicaments. It is effective against primary infections. However, its effectiveness against Enterococcus (E.) faecalis and Candida (C.) albicans is controversial. On the other hand, chlorhexidine (CHX) is a potent agent against E. faecalis and C. albicans. For this reason, the combination of Ca(OH)_2_ and CHX has been suggested as an intracanal medicament. The purpose of this article was to review antimicrobial efficacy of Ca(OH)_2_, CHX as well as their combination.

## Introduction

Micro-organisms play an essential role in the development and perpetuation of pulp and periapical diseases [1][2][3]. Elimination of micro-organisms from infected root canal systems (RCS) is a complicated task. Numerous measures have been described to reduce the number of micro-organisms from the root canal system, including the use of various mechanical instrumentation techniques, irrigation regimes and intra-canal medicaments. There is no definitive evidence in literature to show that mechanical instrumentation alone will predictably result in bacteria-free RCS’s, which is not surprising given the complex anatomy of the RCS [4]. On the contrary, there is both in vitro and clinical evidence that mechanical instrumentation leaves significant portions of the root canal walls untouched [5]. Hence complete elimination of bacteria from the RCS by instrumentation alone is unlikely to be achieved [6]. It is assumed, but not demonstrated, that any pulp tissue left in the RCS can serve as a source of nutrient for bacteria. This however is likely to be for a very short time as any remnant pulp tissue is likely to necrose and be digested by the bacteria within 1-2 months, depending on whether the canal is open to the oral environment or not [7]. Furthermore, tissue remnants may impede the antimicrobial effects of root canal irrigants and medicaments. Therefore, some form of chemical irrigation and disinfection is necessary to remove tissue and other debris from the RCS and to kill remaining micro-organisms. Chemical treatment of the RCS can be arbitrarily divided into several phases, namely irrigants, rinses, and inter-appointment medicaments.

### Definition of a medicament

Medicament is an effective antimicrobial agent which is placed inside the root canal between treatment appointments in order to destroy remaining micro-organisms and prevent the growth of any new arrivals [8].

### Calcium hydroxide

#### Chemical composition and activity

Calcium hydroxide [Ca(OH)_2_] was originally introduced to the field of endodontics by Herman as a direct pulp-capping agent [9]. It is a white odourless powder with the formula Ca(OH)_2_, and a molecular weight of 74.08. It has low solubility in water (about 1.2 gL^-1^ at 25ºC), which decreases as the temperature rises [10]. It has been shown that it’s the dissociation coefficient of Ca(OH)_2_ of 0.17 that permits a slow, controlled release of both calcium and hydroxyl ions. The low solubility is a good clinical characteristic as a long period is necessary before it becomes soluble in tissue fluids when in direct contact with vital tissues [11]. It has a high pH (about 12.5-12.8) and is insoluble in alcohol. The material is chemically classified as a strong base, it main actions come from the ionic dissociation of Ca^2+^ and OH^-^ ions and their effect on vital tissues, generating the induction of hard tissue deposition and being antibacterial [9]. Estrela and Pesce [12] chemically analyzed the liberation of calcium and hydroxyl ions from Ca(OH)_2_ pastes with vehicles of different acid-base and hydrosolubility characteristics by means of conductometer analysis of their solutions in connective tissue of a dog. The liberation of hydroxyl ions from the pastes can be demonstrated by the liberation of calcium ions and hydroxyl ions and the molecular weight of Ca(OH)_2_. In Ca(OH)_2_, the proportion of hydroxyl ions to calcium ions is 45.89% to 54.11%. Ca(OH)_2_ in water has a thixotropic behavior. This means it will be very fluid when agitated [11]. When Ca(OH)_2_ is exposed to carbon dioxide (CO_2_) or carbonate ions (CO_3_^-^) in biological tissue, the dissociation of the chemical leads to formation of calcium carbonate (CaCO_3_) and an overall consumption of Ca^2+^ ions. However, it has been shown that after 30 days of exposure to carbon dioxide, six preparations of Ca(OH)_2_ still maintained a purportedly bactericidal pH within the root canal [10]. Estrela and Pesce [13] also showed that when saline vehicles were used with Ca(OH)_2_ paste, the rate of formation of calcium carbonate was practically unaltered after 30 days and up to 60 days.

#### Mechanism of antimicrobial action

Antimicrobial activity of Ca(OH)_2_ is related to the release of hydroxyl ions in an aqueous environment. Their lethal effects on bacterial cells are probably due to the following mechanisms: damage to the bacterial cytoplasmic membrane; protein denaturation; and damage to the DNA.

Although scientific evidence suggests that the three mechanisms may occur, it is difficult to establish, in a chronological sense, which is the main mechanism involved in the death of bacterial cells after exposure to a strong base.

Adjustment of intracellular pH is influenced by different cellular processes such as: a) cellular metabolism, b) alterations in shape, mobility, adjustment of transporters and polymerization of cytoskeleton components, c) activation of cellular proliferation and growth, d) conductivity and transport through the membrane, and e) isosmotic cellular volume. Thus, many cellular functions can be affected by pH, including the enzymes that are essential to cellular metabolism [14].

#### Antimicrobial activity

Calcium hydroxide exerts antibacterial effects in the root canal system as long as the high pH is retained. In their in vivo study, Byström et al. found that root canals treated with Ca(OH)_2_ had fewer bacteria than those treated with camphorated phenol or camphorated monochlorophenol [15]. They attributed this to the fact that Ca(OH)_2_ can be packed into the root canal system allowing hydroxyl ions to be released over a long period of time. Stevens and Grossman [16] also showed Ca(OH)_2_ to be effective in preventing the growth of microorganisms but to a limited extent when compared to CMCP, stressing the necessity of direct contact to achieve antibacterial effect. Sjogren et al. demonstrated that a 7-day usage of Ca(OH)_2_ medicament was sufficient to reduce canal bacteria to a level that gave a negative culture [17]. In a study to evaluate the effect of electrophoretically activated Ca(OH)_2_ on bacterial viability in dentinal tubules, Lin et al. found that treatment with electrophoresis was significantly more effective than pure Ca(OH)_2_ in depths of 200-500 micrometres [18]. Specimens treated with electrophoretically activated Ca(OH)_2_ showed no viable bacteria in dentinal tubules to a depth of 500 micrometres from the root canal space within 7 days. Portenier et al. showed that E. faecalis cells in their exponential growth phase were the most sensitive to Ca(OH)_2_ paste and were killed between 3 sec and 10 min [19]. Cells in stationary phase were more resistant and living cells could be recovered in 10 min. However, cells in starvation phase were the most resistant and were not totally eliminated during the 10-min test period.

By contrast, several studies have attested to the inefficacy of Ca(OH)_2_ in eliminating bacterial cells. DiFiore et al. found that Ca(OH)_2_ had no antibacterial effect as a paste, or as the commercial preparation Pulpdent when used against S. Sanguis [20]. These findings were confirmed by a further study [21].

Haapasalo and Ørstavik reported that a Ca(OH)_2_ paste (Calasept) failed to eliminate, even superficially, E. faecalis in dentinal tubules [22]. Safavi et al. demonstrated that Enterococcus (E.) faecium remained viable in dentinal tubules after relatively extended periods of Ca(OH)_2_/saline mixture treatment [23]. Ørstavik and Haapasalo observed that Ca(OH)_2_ can take up to 10 days to disinfect dentinal tubules infected by facultative bacteria [24]. Siqueira and Uzeda demonstrated that Ca(OH)_2_ mixed with saline was ineffective in eliminating E. faecalis and E. faecium inside dentinal tubules even after 1 week of contact [25]. Weiger et al. showed that the viability of E. faecalis in infected root dentine was not affected by Ca(OH)_2_ [26]. In a polymerase chain reaction (PCR) study to evaluate the effect of root canal obturation with or without prior Ca(OH)_2_ or 2% chlorhexidine (CHX) on the persistence of bacterial DNA in infected dentinal tubules, Cook et al. found that 2% CHX treatment followed by obturation was more effective in removing E. faecalis DNA than placement of Ca(OH)_2_ or immediate obturation [27]. Ballal et al. found that in failed root canal treatments, 2% CHX gel may be a more effective intracanal medicament than Ca(OH)_2_ paste against E. faecalis [28]. Krithikadatta et al. showed that as an intracanal medicament, %2 CHX gel alone was more effective against E. faecalis when compared to Ca(OH)_2_ [29].

Waltimo et al. found that C. albicans cells were highly resistant to Ca(OH)_2_ [30]. Siqueira et al. investigated the antifungal ability of several medicaments against C. albicans, C. glabrata, C. guilliermondii, C. parapsilosis, and S. cerevisiae [31]. Whereas the paste of Ca(OH)_2_ in CPMC/glycerin showed the most pronounced antifungal effects, Ca(OH)_2_ in glycerin or CHX and CHX in detergent also showed antifungal activity that was much lower than the paste of Ca(OH)_2_ in CPMC/glycerin. In another study, the in vitro susceptibility of C. albicans to various irrigants and medicaments showed that NaOCl, hydrogen peroxide, and CHX digluconate were effective against C. albicans even when significantly diluted [32]. Aqueous Ca(OH)_2_ had no activity. When maintained in direct contact with C. albicans cells, Ca(OH)_2_ paste and CPMC were effective in killing this microorganism. The antifungal effectiveness of CPMC was also shown by a study that investigated the effectiveness of several intracanal medications on C. albicans harvested inside root canals, observing that CPMC was the most effective, followed by Ca(OH)_2_/CPMC paste.

A further study evaluated the effectiveness of 4 intracanal medicaments in disinfecting the root dentin of bovine teeth experimentally infected with C. albicans. Infected dentin cylinders were exposed to 5 different medications: Ca(OH)_2_/glycerin, Ca(OH)_2_/0.12% CHX digluconate, Ca(OH)_2_/CPMC/glycerin, and 0.12% CHX digluconate/zinc oxide. Specimens were left in contact with the medicaments for 1 hour, 2 days, and 7 days. The specimens treated with Ca(OH)_2_/CPMC/glycerin paste or with CHX/zinc oxide paste were completely disinfected after 1 hour of exposure. Ca(OH)_2_/glycerin paste only consistently eliminated C. albicans infection after 7 days of exposure. Ca(OH)_2_ mixed with CHX was ineffective in disinfecting dentin even after 1 week of medicament exposure. Of the medicaments tested, the Ca(OH)_2_/CPMC/glycerin paste and CHX digluconate mixed with zinc oxide were the most effective in eliminating C. albicans cells from dentinal specimens.

### Chlorhexidine gluconate

#### Structure and mechanism of action

Chlorhexidine consists of two symmetric 4-cholorophenyl rings and two biguanide groups connected by a central hexamethylene chain [33]. CHX is a positively charged hydrophobic and lipophilic molecule that interacts with phospholipids and lipopolysaccharides on the cell membrane of bacteria and then enters the cell through some type of active or passive transport mechanism [34]. Its efficacy is due to the interaction of the positive charge of the molecule and the negatively charged phosphate groups on the microbial cell walls [34], thereby altering the cells' osmotic equilibrium. This increases the permeability of the cell wall, which allows the CHX molecule to penetrate into the bacterial cell. CHX is a base and is stable as a salt. The most common oral preparation, CHX gluconate, is water-soluble and, at physiologic pH, it readily dissociates and releases the positively charged CHX component [34]. At low concentration (such as 0.2%), low molecular weight substances-specifically potassium and phosphorous-will leak out. A higher concentrations (e.g. 2%), CHX is bactericidal and precipitation of cytoplasmic contents occurs which results in cell death [34].

#### Antimicrobial activity

Delany et al. evaluated 0.2% CHX-gluconate in infected root canals [35]. Bacteriologic samples were obtained before, during, immediately after and 24 hours after instrumentation, irrigation and medication either with CHX-gluconate or with sterile saline. There was a highly significant reduction in the number of microorganisms in the CHX-treated specimens after instrumentation and irrigation. Oncag et al. evaluated the antibacterial properties of 5.25% sodium hypochlorite (NaOCl), 2% CHX and 0.2% CHX plus 0.2% cetrimide [Cetrexidin (GABA Vebas, San Giuliano Milanese, Italy)] after 5 minutes and 48 hours in extracted human teeth after the canals had been infected by E. faecalis [36]. The 2% CHX and Cetrexidin were significantly more effective on E. faecalis than the 5.25% NaOCl at both time periods.

Zamany et al. examined the effects of adding a 2% CHX rinse to the conventional treatment protocol [37]. Their results showed that cultivable bacteria were retrieved at the conclusion of the first visit in one of the CHX cases whereas seven of the 12 control cases without CHX showed growth; this difference was statistically significant. Siqueira et al. compared the effectiveness of 2.5% sodium hypochlorite and 0.12% CHX as irrigants in reducing the cultivable bacteria in infected root canal systems with apical periodontitis [38]. They found that the two solutions had comparable effects in eliminating bacteria and they suggested that both could be used as irrigants. This result is supported by other studies [39]

In a randomized clinical trial, Manzur et al. assessed the antibacterial efficacy of intracanal medication with Ca(OH)_2_, 2% CHX gel, and a combination of both (Ca(OH)_2_/CHX) in teeth with chronic apical periodontitis [40]. Findings revealed that the antibacterial efficacies of Ca(OH)_2_, CHX, and a mixture of Ca(OH)_2_/CHX were comparable.

Zerella et al. investigated the effect of a slurry of Ca(OH)_2_ mixed in aqueous 2% CHX versus aqueous Ca(OH)_2_ alone on the disinfection of the root canal system of root filled teeth that required root canal re-treatment because the canals had become infected again [41]. Their results indicated that a mixture of 2% CHX and a Ca(OH)_2_ slurry is as efficacious as aqueous Ca(OH)_2_ on the disinfection of infected root filled teeth.

Tanomaru et al. evaluated the effect of biomechanical preparation with 5% NaOCl, 2% CHX and physiological saline irrigating solutions and Ca(OH)_2_ dressing in the root canals of dogs’ teeth that contained bacterial endotoxin [42]. They found that biomechanical preparation with the irrigating solutions did not inactivate the endotoxin but the Ca(OH)_2_ intracanal dressing did inactivate the effects induced by the endotoxin in vivo.

Waltimo et al. evaluated the susceptibility of seven strains of C. albicans to four disinfectants, namely IKI, CHX-acetate (0.5%), NaOCl (5% and 0.5%), and Ca(OH)_2_ [30]. All C. albicans strains tested showed similar susceptibility to these medicaments. They were highly resistant to Ca(OH)_2_ but the NaOCl and IKI killed all cells within 30 seconds and the CHX-acetate showed complete killing after 5 minutes. Combinations of disinfectants were either equally or less effective than the more effective component of the pair tested.

Siqueira et al. also investigated the antifungal activity of several medicaments against C. albicans, C. glabrata, C. guilliermondii, C. parapsilosis, and S. cerevisiae [34]. Ca(OH)_2_ mixed with CPMC/glycerin as a paste showed the most pronounced antifungal effects. Ca(OH)_2_ in glycerin, Ca(OH)_2_ with CHX, and CHX in detergent had less antifungal activity. Ferguson et al. sought to determine the in vitro susceptibility of C. albicans to various irrigants and medicaments [32]. The minimum inhibitory concentrations of NaOCl, hydrogen peroxide, CHX-digluconate, and aqueous Ca(OH)_2_ were determined. Their results revealed that NaOCl, hydrogen peroxide, and CHX-digluconate were effective against C. albicans even when significantly diluted. However, aqueous Ca(OH)_2_ had no anti-fungal activity.

On the whole, it seems that when used in identical concentrations, the antibacterial effects of CHX and NaOCl are similar. CHX is an effective antifungal agent and its efficacy is significantly less than NaOCl.

### CHX and Calcium hydroxide

#### Chemical viewpoints

Combined use of CHX and Ca(OH)_2_ in the root canal may generate excessive reactive oxygen species, which may potentially kill various root canal pathogens [43]. Furthermore, it has been demonstrated that the alkalinity of Ca(OH)_2_ when mixed with CHX remained unchanged [44].

Antimicrobial activity

In a study by Almyroudi et al., all of the CHX formulations used, including a CHX/CH 50:50 mix, were efficient in eliminating E. faecalis from the dentinal tubules with a 1% CHX gel working slightly better than the other preparations [45]. These findings were corroborated by Gomes et al. [46] in bovine dentine and Schafer and Bossmann [47] in human dentine where 2% CHX gel had greater activity against E. faecalis, followed by CHX/CH and then CH used alone.

In a study using agar diffusion, researchers could not demonstrate any additive antibacterial effect by mixing CH powder with 0.5% CHX and they showed that the CHX had a reduced antibacterial action [44]. However, CH did not lose its antibacterial properties in such a mixture. This may be due to the deprotonation of CHX at a pH greater than 10, which reduces its solubility and alters its interaction with bacterial surfaces as a result of the altered charge of the molecule. In an in vitro study using human teeth, Ercan et al. [48] showed 2% CHX gel was the most effective agent against E. faecalis inside dentinal tubules, followed by a CH/2% CHX mix, whilst CH alone was totally ineffective, even after 30 days. The 2% CHX gel was also significantly more effective than the CH/2% CHX mix against C. albicans at seven days, although there was no significant difference at 15 and 30 days. CH alone was completely ineffective against C. albicans. These results were further validated by another in vivo study using primary teeth. A 1% CHX-gluconate gel, both with and without CH, was more effective against E. faecalis than CH alone over a 48-hour period [49].

Schafer and Bossnamm reported that 2% CHX-gluconate was significantly more effective against E. faecalis than CH used alone, or a mixture of the two [47]. This was also confirmed by Lin et al. [50] although in a study by Evans et al. [51] using bovine dentine, 2% CHX with CH was shown to be more effective than CH in water. In an animal study, Lindskog et al. reported that teeth dressed with CHX for four weeks had reduced inflammatory reactions in the periodontium (both apically and marginally) and less root resorption [52]. Waltimo et al. [30] reported that 0.5% CHX-acetate was more effective at killing C. albicans than saturated CH, while CH combined with CHX was more effective than CH used alone. The high pH of CH was unaffected when combined with CHX in this study. Another study evaluated the effectiveness of 2% CHX solution mixed with CH against C. albicans and found that combining these agents was beneficial [53].

## Conclusions

1.Antimicrobial activity of Ca(OH)_2_ is related to the release of hydroxyl ions in an aqueous environment.

2.Ca(OH)_2_ seems to be ineffective against E. faecalis and C. albicans, and therefore has no efficacy in retreatment cases.

3.CHX has a wide range of activity against both gram positive and Gram negative bacteria.

4.CHX is an effective antifungal agent especially against C. albicans.

5.Mixing CHX with Ca(OH)_2_ may enhance its antimicrobial activity.
